# Summer, Malaria, &c.

**Published:** 1881-08

**Authors:** 


SUMMER, MALARIA, &c.—The season has arrived for the summer exodus of many of the dwellers in our cities and large towns, and malaria is beginning to be talked about, and it may be, dreamed about, by those anxious to hie themselves to some one of the hundreds of resorts where people most do congregate for health or pleasure.

Relaxation from business, and the mental and physical worry and fatigue incident to all bread-winning avocations, certainly tend in a great measure towards restoring strength, and even health, in certain cases, and consequently to the prolongation of human life; and when good bracing air, pure water, wholesome food, with pleasant temperature, and agreeable associations are added, some pecuniary sacrifices should be made to procure the comfort and benefits which such surroundings afford.

But when enjoying the pleasures and advantages alluded to above, one is conscious of escaping the old and dreaded summer enemy malaria, they poseess a keener zest, and the time and money, necessary to their procurement, are, or should be ungrudgingly spent.

